# Pharmacological Stimulation of Soluble Guanylate Cyclase Counteracts the Profibrotic Activation of Human Conjunctival Fibroblasts

**DOI:** 10.3390/cells13040360

**Published:** 2024-02-18

**Authors:** Bianca Saveria Fioretto, Irene Rosa, Elena Andreucci, Rita Mencucci, Mirca Marini, Eloisa Romano, Mirko Manetti

**Affiliations:** 1 Section of Anatomy and Histology, Department of Experimental and Clinical Medicine, University of Florence, Largo Brambilla 3, 50134 Florence, Italy; biancasaveria.fioretto@unifi.it (B.S.F.); irene.rosa@unifi.it (I.R.); mirca.marini@unifi.it (M.M.); 2 Section of Experimental Pathology and Oncology, Department of Experimental and Clinical Biomedical Sciences “Mario Serio”, University of Florence, Viale Morgagni 50, 50134 Florence, Italy; e.andreucci@unifi.it; 3 Eye Clinic, Careggi Hospital, Department of Neurosciences, Psychology, Pharmacology and Child Health (NEUROFARBA), University of Florence, Largo Brambilla 3, 50134 Florence, Italy; rita.mencucci@unifi.it; 4 Section of Internal Medicine, Department of Experimental and Clinical Medicine, University of Florence, Largo Brambilla 3, 50134 Florence, Italy; eloisa.romano@unifi.it; 5 Imaging Platform, Department of Experimental and Clinical Medicine, University of Florence, Largo Brambilla 3, 50134 Florence, Italy

**Keywords:** conjunctival fibroblasts, myofibroblasts, conjunctival fibrosis, TGFβ1, soluble guanylate cyclase stimulation

## Abstract

Conjunctival fibrosis is a serious clinical concern implicated in a wide spectrum of eye diseases, including outcomes of surgery for pterygium and glaucoma. It is mainly driven by chronic inflammation that stimulates conjunctival fibroblasts to differentiate into myofibroblasts over time, leading to abnormal wound healing and scar formation. Soluble guanylate cyclase (sGC) stimulation was found to suppress transforming growth factor β (TGFβ)-induced myofibroblastic differentiation in various stromal cells such as skin and pulmonary fibroblasts, as well as corneal keratocytes. Here, we evaluated the in vitro effects of stimulation of the sGC enzyme with the cell-permeable pyrazolopyridinylpyrimidine compound BAY 41-2272 in modulating the TGFβ1-mediated profibrotic activation of human conjunctival fibroblasts. Cells were pretreated with the sGC stimulator before challenging with recombinant human TGFβ1, and subsequently assayed for viability, proliferation, migration, invasiveness, myofibroblast marker expression, and contractile properties. Stimulation of sGC significantly counteracted TGFβ1-induced cell proliferation, migration, invasiveness, and acquisition of a myofibroblast-like phenotype, as shown by a significant downregulation of *FAP*, *ACTA2*, *COL1A1*, *COL1A2*, *FN1*, *MMP2*, *TIMP1*, and *TIMP2* mRNA levels, as well as by a significant reduction in α-smooth muscle actin, N-cadherin, COL1A1, and FN-EDA protein expression. In addition, pretreatment with the sGC stimulator was capable of significantly dampening TGFβ1-induced acquisition of a contractile phenotype by conjunctival fibroblasts, as well as phosphorylation of Smad3 and release of the proinflammatory cytokines IL-1β and IL-6. Taken together, our findings are the first to demonstrate the effectiveness of pharmacological sGC stimulation in counteracting conjunctival fibroblast-to-myofibroblast transition, thus providing a promising scientific background to further explore the feasibility of sGC stimulators as potential new adjuvant therapeutic compounds to treat conjunctival fibrotic conditions.

## 1. Introduction

The conjunctiva is the thin and almost completely transparent mucous membrane covering the inner surface of the eyelids and the anterior surface of the eyeball, with the exception of the cornea [1]. In the space between the eyelids and the eyeball, it folds and covers the upper and lower fornices, allowing the bulb to have freedom of movement [1]. Histologically, the conjunctiva is composed of a lining epithelium, with epithelial cells arranged in 2–5 layers, an underlying fibroblast-rich stroma, and a glandular system made up of goblet cells containing mucin granules and producing the mucin component of the tear film [1]. The conjunctiva has the main function of providing a coating to protect the eye from foreign bodies and infections. Furthermore, it helps to maintain the tear film, facilitates the sliding of the eyelids during blinking phases, and exerts immunological surveillance [1]. The conjunctiva can be affected by various infectious, inflammatory, and degenerative pathological processes, including conjunctivitis, immune-mediated disorders such as Stevens-Johnson syndrome, toxic epidermal necrolysis or pemphigoid [2,3], and pterygium [4], which ultimately lead to conjunctival scarring. Another frequent cause of conjunctival fibrosis is represented by the failure of both filtration surgery (trabeculectomy) and prolonged use of ophthalmic pharmaceuticals for the treatment of glaucoma, a disease characterized by a rise in intraocular pressure leading to damage to the optic nerve and consequent visual field defects [5,6,7]. Fibrotic scarring is indeed a severe trabeculectomy side effect, as it has been demonstrated that, after surgery, conjunctival fibroblasts start to proliferate and release collagen and other components of the extracellular matrix (ECM), with consequent formation of fibrotic tissue that impedes the normal flow of aqueous humor [6,7,8,9]. Postoperative inflammation may also intensify the wound healing process that follows incisional surgery, further fostering scar formation and augmenting the risk of fibrosis and impairment of filtration bleb functionality [6,10,11,12]. Moreover, long-term use of anti-glaucoma eye drops is known to promote ocular surface changes that contribute to conjunctival inflammation and fibrosis [6,10,13,14].

Among the molecular mechanisms underlying conjunctival fibrosis, transforming growth factor β (TGFβ) has been demonstrated to play a key pathological role by increasing fibroblast proliferation and promoting their conversion into myofibroblasts, ultimately leading to abnormal deposition of ECM proteins and consequent tissue scarring [15,16,17,18,19,20,21,22,23,24].

At present, management of conjunctival fibrosis is rather limited and mainly based on the inhibition of the TGFβ-signaling pathway by means of small molecule inhibitors or viral transfection [3,9,24]. However, since these therapeutic strategies have so far demonstrated poor safety and scarce clinical benefits, the discovery of new potential drugs able to prevent or counteract conjunctival fibrosis becomes of crucial importance. In this context, increasing literature reported antifibrotic effects exerted by the stimulation of soluble guanylate cyclase (sGC), an enzyme that, after binding of nitric oxide (NO) to its prosthetic heme group, catalyzes the production of cyclic guanosine monophosphate (cGMP), thus regulating different biological processes, including cell growth/proliferation and ECM synthesis [25,26]. Of note, sGC stimulator compounds are able to bind to the native heme-containing reduced form of sGC and stimulate the enzyme both in synergy with and independently of NO raising intracellular cGMP levels [25]. In particular, the selective sGC stimulators BAY 60-277, BAY 41-2272, and riociguat have been shown to ameliorate hepatic, cardiac, and dermal fibrosis in vivo [27,28,29,30,31,32], while treatment with the BAY 41-2272 compound has inhibited TGFβ1-mediated cardiac, pulmonary, and dermal fibroblast-to-myofibroblast differentiation in vitro [27,30,33,34,35]. Moreover, another sGC stimulator, MK-2947, was found to dampen endothelial-to-myofibroblast transition [36]. Notably, our group has recently demonstrated that sGC stimulation with the BAY 41-2272 compound could reduce TGFβ1-induced myofibroblastic differentiation of human corneal stromal cells/keratocytes, suggesting its potential utility for the treatment of corneal fibrotic scarring [37]. Based on these premises, the current in vitro study was designed to investigate whether pharmacological stimulation of the sGC enzyme could also prove effective in counteracting profibrotic activation of human conjunctival fibroblasts.

## 2. Materials and Methods

### 2.1. In Vitro Culture of Human Conjunctival Fibroblasts

All steps were performed in sterile conditions, under a biological hood. Three commercial lines of healthy human adult conjunctival fibroblasts (HConF) purchased from Innoprot (P10876; Innoprot, Derio, Spain) were grown at 37 °C in a 5% CO_2_ incubator in 4.5 g/L glucose Dulbecco’s Modified Eagle Medium (DMEM; 11-965-092; Thermo Fisher Scientific, Waltham, MA, USA) supplemented with 10% fetal bovine serum (FBS; ECS1104L; Euroclone, Milan, Italy), L-Glutamine (ECB3000D; Euroclone) and a solution containing 100 µg/mL streptomycin and 100 U/mL penicillin (ECB3001D; Euroclone). Once at confluence, cells were collected and plated on different cell culture supports according to the specific assays. The culture medium was changed every three days and cells were assayed no later than the fifth passage.

### 2.2. Stimulation of Human Conjunctival Fibroblasts

Before treatments, conjunctival fibroblasts were cultured up to 70% confluence, washed with medium without FBS, and then left overnight in basal DMEM additioned with 2% FBS (starvation medium). Cells were subsequently pretreated for 2 h with cell-permeable pyrazolopyridinylpyrimidine BAY 41-2272 compound as an sGC stimulator (B8810; Sigma-Aldrich, St. Louis, MO, USA) at 1 µM or 10 µM concentrations before the addition of 10 ng/mL recombinant human TGFβ1 (PeproTech, Rocky Hill, NJ, USA). The 1 µM and 10 µM BAY 41-2272 concentrations were chosen on the basis of previous in vitro studies that used the same compound to counteract TGFβ1-induced myofibroblastic transition of different stromal cell types, including primary human dermal fibroblasts [27,33], human lung fibroblasts [34], and human corneal keratocytes [37]. Cells were also stimulated with TGFβ1 alone. The BAY 41-2272 compound solution was prepared in dimethyl sulfoxide, whose final concentration did not exceed 0.1%. After stimulation for 48 h, conjunctival fibroblasts were analyzed for cell viability, proliferation, invasiveness, chemotactic activity, and mRNA expression, whereas protein expression and the capability to contract a collagen matrix were evaluated after 72 h. The culture supernatant of 72 h-challenged fibroblasts was harvested, centrifuged (1500 rpm, 10 min), and stored at −20 °C.

### 2.3. Cyclic Guanosine Monophosphate Assay

Intracellular cGMP levels were measured after 24 h using a colorimetric cGMP complete enzyme-linked immunosorbent assay kit (ADI-900-164; Enzo Life Sciences, Farmingdale, NY, USA) according to the manufacturer’s protocol.

### 2.4. Annexin V/Propidium Iodide Flow Cytometer Assay

Conjunctival fibroblasts were maintained in a starvation medium overnight and subsequently stimulated for 48 h, as described above. Cells were then collected with Accutase (ECB3056D; Euroclone), transferred in flow cytometer tubes, and subjected to an annexin V/propidium iodide flow cytometer assay, as described in detail previously [38]. The specimens were analyzed with a BD FACS Canto II flow cytometer (BD Biosciences, Franklin Lakes, NJ, USA). Each cell line was tested in triplicate. At least 10,000 events were collected for each flow cytometer tube.

### 2.5. Cell Proliferation Assay

Conjunctival fibroblasts were seeded onto 96-well plates in complete DMEM. The day after, fibroblasts were washed, maintained in a starvation medium for a further 24 h, and then challenged for 48 h in starvation medium additioned with the sGC stimulator BAY 41-2272 (1 µM or 10 µM), recombinant human TGFβ1, or TGFβ1 administered 2 h after pretreatment with BAY 41-2272 (1 µM or 10 µM). The proliferation of conjunctival fibroblasts was assessed by evaluating cell metabolism with the WST-1 assay (5015944001; Merck, Darmstadt, Germany). The proliferation of each cell line was measured in triplicate. Results were presented as a percentage of increase/decrease in proliferative rate over the basal response (i.e., fibroblasts maintained in starvation medium).

### 2.6. Cell Morphology and Confluency Assessment

To assess cell morphology and cell confluency, ×10 phase-contrast images were captured under a Mateo TL RUO microscope equipped with a confluency module (Leica Microsystems, Mannheim, Germany). Cell confluency was evaluated after plating cells in a T75 flask at a density of 5 × 10^5^ followed by stimulation for 48 h, as described above. The arrangement of the F-actin cytoskeleton was visualized by Alexa 488-conjugated phalloidin (1:40 dilution; Invitrogen, Carlsbad, CA, USA) staining, while focal adhesions were immunostained by using a mouse monoclonal anti-vinculin antibody (1:100 dilution; V9131; Sigma-Aldrich). Nuclear counterstaining was performed with 4′,6-diamidino-2-phenylindole (DAPI).

### 2.7. In Vitro Wound Healing Assay

Conjunctival fibroblasts were cultured in 6-well plates in complete DMEM. After reaching 80–90% confluency, fibroblasts were washed and maintained in a starvation medium overnight. Then, the culture medium was removed, and the cell monolayer was scratched by means of a sterile 200-μL pipette tip to obtain a wound of ~1 mm width. Detached cells were removed by gently washing with basal DMEM, and cultures were then treated for 24 h with starvation medium additioned with BAY 41-2272 (10 µM), recombinant human TGFβ1, or TGFβ1 administered 2 h after pretreatment with BAY 41-2272 (10 µM). No inhibitors of mitosis were used. Wound-healing ability was determined by taking phase-contrast images both immediately after scratching and 24 h later under a Mateo TL RUO microscope (Leica Microsystems) by using a ×4 objective. Pictures captured at the beginning (0 h) and after 24 h were compared to measure the wound closure rate. Every cell line was assayed in triplicate for each experimental condition.

### 2.8. Chemotaxis and Chemoinvasion Assays

Cell migration (chemotaxis) was assessed by the Boyden chamber method using 24-well culture plates, in which inserts equipped with a polyethylene terephthalate membrane filter (83.3932.800; Sarstedt, Nümbrecht, Germany) were placed to divide the migration chamber into an upper and a lower compartment. Recombinant human TGFβ1 (10 ng/mL) was added as a chemotactic factor in the lower wells, whereas 25 × 10^3^ conjunctival fibroblasts previously grown for 48 h in culture plates at basal condition or in the presence of BAY 41-2272 (10 µM), recombinant human TGFβ1, or TGFβ1 preceded by BAY 41-2272 (10 µM) were collected, resuspended in starvation medium, and seeded on the upper wells. To exclude chemokinesis, in some experimental points TGFβ1 was not added to the starvation medium in the inferior compartment as a negative control. Every experimental point was carried out in triplicate for each cell line. The following day, a cotton swab was used to mechanically wipe off non-migrated fibroblasts from the upper side of the filters, whereas migrated fibroblasts, adherent to the lower filter sides, were fixed and successively stained with a Diff-Quik dye kit (9990700; Epredia, Portsmouth, New Hampshire). Membranes were subsequently washed, removed from the inserts, and then mounted on glass microscope slides. Migrated cells were finally photographed with a Leica DFC310 FX 1.4-megapixel digital camera installed onto a Leica DM4000 B microscope (Leica Microsystems). Migrated fibroblasts were counted with open-source CellCounter software (online at https://bitbucket.org/linora/cellcounter/downloads/, accessed on 9 October 2023). The chemoinvasion assay was performed using the same experimental procedure, by employing filters precoated overnight with 0.25 µg/µL Geltrex (A1413202; Thermo Fisher Scientific) before cell seeding.

### 2.9. Quantitative Real-Time PCR

After stimulation for 48 h, conjunctival fibroblasts were collected, and the RNA content, purified with the RNeasy Micro Kit (74004; Qiagen, Milan, Italy), was quantified with a NanoDrop 8000 Spectrophotometer (Thermo Fisher Scientific). cDNA synthesis and SYBR Green real-time PCR were performed, as described in detail previously [37]. Details of oligonucleotide primer pairs (QuantiTect primer assays; Qiagen) are provided in Table 1. The endogenous control used for normalization was 18S ribosomal RNA (Hs_RRN18S_1_SG; QT00199367; Qiagen). Threshold cycle (Ct) and comparative Ct methodologies for relative quantification were used to calculate gene expression differences. Every gene assay was done in triplicate for each of the three conjunctival fibroblast lines.

### 2.10. Western Blotting

Cellular pellets were collected from conjunctival fibroblasts stimulated for 72 h. Proteins were extracted by lysing cells in RIPA lysis buffer (89901; Thermo Fisher Scientific) additioned with a complete protease inhibitor cocktail (11697498001; Roche, Basilea, Switzerland), 1 mM sodium orthovanadate, and 1 mM NaF, followed by sonication. The solution was then subjected to protein content quantification by Bradford’s method. Proteins (40 µg) were processed, as described in detail elsewhere [37], and subsequently blotted onto nitrocellulose membranes by using Trans-Blot Turbo Mini 0.2 µm Nitrocellulose Transfer Packs (#1704158; Bio-Rad, Hercules, CA, USA) and the Trans-Blot Turbo transfer system instrument (Bio-Rad). The Western blotting analysis was carried out following previously published protocols [37], and protein bands were revealed by the ChemiDoc Touch Imaging System (Bio-Rad). Details of the primary antibodies used are presented in Table 2. Band densitometry was done with ImageJ software (NIH, Bethesda, MD, USA; online at http://rsbweb.nih.gov/ij, accessed on 16 October 2023).

### 2.11. Fluorescence Immunocytochemistry

Conjunctival fibroblasts were seeded on glass microscope coverslips in 6-well culture plates and stimulated for 72 h, as described in Section 2.2. Once fixed with 3.7% buffered paraformaldehyde, cells were permeabilized with 0.1% Triton X-100 in PBS for 10 min at room temperature, blocked with 1% bovine serum albumin in PBS for 1 h at room temperature, and finally incubated overnight at 4 °C with mouse monoclonal anti-α-smooth muscle actin (α-SMA) (1:100; ab7817; Abcam, Cambridge, UK) and rabbit monoclonal anti-COL1A1 (1:300; #39952; Cell Signaling Technology, Danvers, MA, USA) primary antibodies. Negative controls were obtained by employing irrelevant isotype- and concentration-matched IgG (Sigma-Aldrich). Alexa Fluor-488- or Rhodamine Red-X-conjugated IgG (1:200; Invitrogen) were then applied to the specimens for 45 min at room temperature as secondary antibodies. After nuclear counterstain with DAPI, coverslips were mounted onto glass microscope slides and immunolabeled cells were visualized and photographed, as described elsewhere [37,38].

### 2.12. Collagen Gel Matrix Contraction Assay

Collagen gel matrix contraction assay was carried out by using a commercial kit (Floating Matrix Model; CBA-5020; Cell Biolabs, San Diego, CA, USA), as previously described in detail [37]. Conjunctival fibroblasts were treated for 72 h, as explained in Section 2.2. Cells were then collected and resuspended in basal medium (4 × 10^6^ cells/mL). Each well of a 24-well cell contraction plate was additioned with 500 µL of a solution obtained by mixing 100 µL of cell suspension and 400 µL of collagen gel matrix solution. Basal medium or medium with the appropriate stimuli was then added on top of each polymerized collagen gel matrix. The assay was executed in triplicate for each cell line. After 24 h, the 24-well cell contraction plate was photographed to measure the area of each collagen gel matrix with ImageJ (NIH). Cell-free gels were included in the assay as negative controls.

### 2.13. Enzyme-Linked Immunosorbent Assay

Interleukin (IL)-1β and IL-6 in culture supernatants of conjunctival fibroblasts were quantified by using quantitative colorimetric sandwich enzyme-linked immunosorbent assay kits (BMS224INSTCE and BMS213INSTCE, respectively; Thermo Fisher Scientific) according to the manufacturer’s instructions. Each culture supernatant was tested in triplicate.

### 2.14. Statistical Analysis

GraphPad Prism 5 software was employed for all statistical analyses. In particular, one-way ANOVA followed by post-hoc Tukey’s test was performed after verifying the normality of data with a Kolmogorov–Smirnov test. For each experimental group, all data are expressed as mean ± standard error of the mean (SEM). Statistical significance was set at *p* < 0.05.

## 3. Results

### 3.1. The sGC Stimulator BAY 41-2272 Has No Effect on Cell Viability and Inhibits TGFβ1-Induced Proliferation of Human Conjunctival Fibroblasts

As shown in Supplementary Figure S1, stimulation of sGC with BAY 41-2272 effectively increased the intracellular levels of cGMP in human conjunctival fibroblasts.

**Figure 1:**
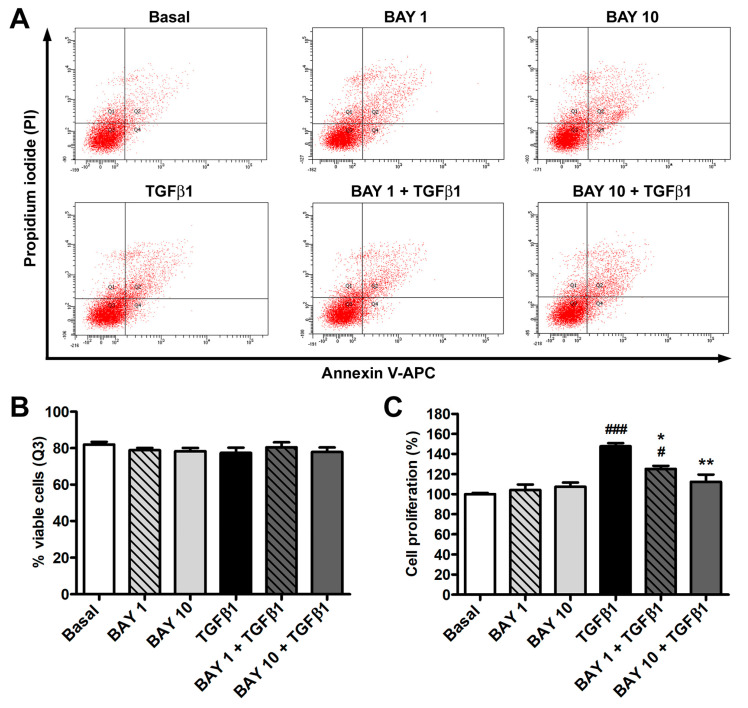
The sGC stimulator BAY 41-2272 does not affect human conjunctival fibroblast viability and inhibits TGFβ1-induced cell proliferation. (**A**) Representative annexin V/PI flow cytometry assay plots of conjunctival fibroblasts at the basal condition and challenged with recombinant human TGFβ1 alone, BAY 41-2272 1 μM or 10 μM alone, or TGFβ1 added 2 h after preincubation with BAY 41-2272 1 µM or 10 µM. The upper left (Q1) quadrant represents annexin V^−^/PI^+^ necrotic cells, the upper right (Q2) quadrant annexin V^+^/PI^+^ late apoptotic cells, the lower left (Q3) quadrant annexin V^−^/PI^−^ viable cells, and the lower right (Q4) quadrant annexin V^+^/PI^−^ early apoptotic cells. (**B**) Mean ± SEM percentage of viable cells (Q3) is reported for each experimental point. (**C**) Cell proliferation assessed with WST-1 colorimetric assay. Cell proliferation at basal condition was set to 100%, and the other results were normalized accordingly. Bars represent the mean ± SEM of triplicate determinations from three cell lines. ### *p* < 0.001 and # *p* < 0.05 vs. basal condition, ** *p* < 0.01 and * *p* < 0.05 vs. TGFβ1 (Tukey’s test). SEM, standard error of the mean; sGC, soluble guanylate cyclase; TGFβ1, transforming growth factor β1.

To exclude any possible side effect exerted by the administration of the sGC stimulator BAY 41-2272, annexin V/propidium iodide flow cytometry was used to evaluate human conjunctival fibroblast viability. As demonstrated by the lack of significant differences in the number of viable, early apoptotic, late apoptotic, and necrotic cells among the different culture conditions, conjunctival fibroblast viability was not affected by treatment with recombinant human TGFβ1, BAY 41-2272 1 μM or 10 μM, either alone or in combination (Figure 1A,B). As far as cell proliferation assessed by WST-1 assay, treatment with TGFβ1 alone led to a significant rise in the proliferative rate, while BAY 41-2272 alone at both concentrations had no effect (Figure 1C). When conjunctival fibroblasts were pretreated with the BAY 41-2272 compound and then challenged with TGFβ1, both BAY 41-2272 concentrations inhibited TGFβ1-induced cell proliferation, but only BAY 41-2272 10 μM was able to maintain the proliferation rate at basal levels (Figure 1C). On the basis of such results, only BAY 41-2272 10 μM was employed for the subsequent experiments.

### 3.2. Cell Morphology and Confluency Assessment

**Figure 2:**
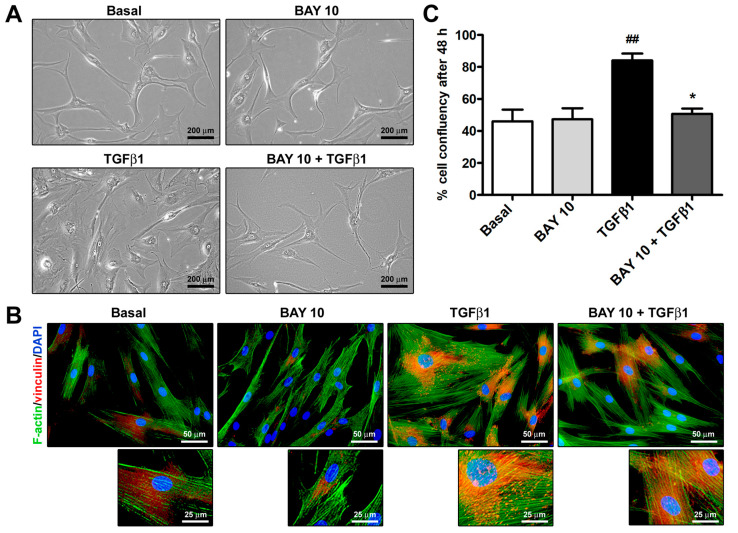
The sGC stimulator BAY 41-2272 inhibits TGFβ1-induced acquisition of a profibrotic myofibroblast-like phenotype by human conjunctival fibroblasts. (**A**) Representative phase-contrast photomicrographs of conjunctival fibroblasts cultured at the basal condition and challenged with recombinant human TGFβ1 or BAY 41-2272 10 µM alone, or TGFβ1 added 2 h after preincubation with BAY 41-2272 10 µM. (**B**) Representative fluorescence photomicrographs of conjunctival fibroblasts stained for F-actin with Alexa 488-conjugated phalloidin (green) and immunostained for vinculin (red). Nuclei are counterstained with DAPI (blue). Higher magnifications are shown in the small bottom panels. Scale bar: 200 μm (**A**), 50 μm (**B**), 25 μm (**B**, bottom panels). (**C**) Quantification of cell confluency 48 h after stimulation. Bars represent the mean ± SEM of triplicate determinations of the percentage of cell confluency. ## *p* < 0.01 vs. basal condition, * *p* < 0.05 vs. TGFβ1 (Tukey’s test). DAPI, 4′,6-diamidino-2-phenylindole; F-actin, filamentous actin; SEM, standard error of the mean; sGC, soluble guanylate cyclase; TGFβ1, transforming growth factor β1.

As shown in Figure 2A, TGFβ1 treatment was able to induce a morphological change in human conjunctival fibroblasts, allowing them to acquire a myofibroblast-like phenotype characterized by larger dimensions and a highly flattened and polygonal-shaped cytoplasm. Moreover, after the challenge with TGFβ1, conjunctival fibroblasts underwent a rearrangement of the F-actin cytoskeleton, which displayed more abundant stress fibers, and there was a formation of vinculin-positive focal adhesions (Figure 2B). Notably, preincubation with BAY 41-2272 10 μM dampened the abovementioned TGFβ1-induced changes in cell morphology and cytoskeletal arrangement (Figure 2A,B). The high confluency percentage of TGFβ1-treated cells further confirmed the ability of TGFβ1 to enhance both cell proliferation and size (Figure 2C). Conversely, cell confluency of conjunctival fibroblast cultures preincubated with BAY 41-2272 10 μM before stimulation with TGFβ1 was similar to that of cultures at basal condition (Figure 2C).

### 3.3. TGFβ1-Induced Wound Healing Ability of Human Conjunctival Fibroblasts Is Reduced by Administration of the SGC Stimulator BAY 41-2272

**Figure 3:**
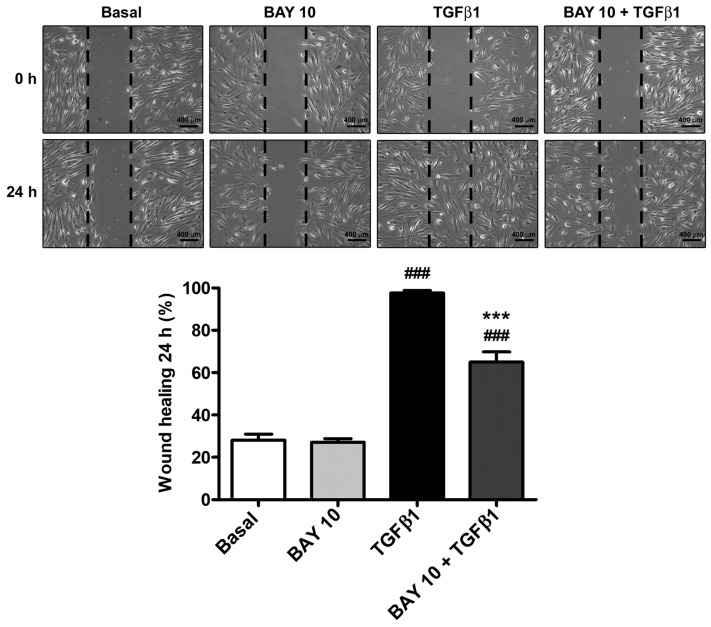
The sGC stimulator BAY 41-2272 significantly reduces the TGFβ1-induced capability of human conjunctival fibroblasts to re-establish monolayer integrity after wounding. Wound-healing ability was assessed in cells at the basal condition and after treatment with TGFβ1, TGFβ1 and BAY 41-2272 10 µM, or BAY 41-2272 alone. Representative phase-contrast photomicrographs of the scratched area at 0 and 24 h are shown. Scale bar: 400 μm. Wound margins are drawn in black. Bars represent the mean ± SEM of triplicate determinations of the percentage of wound healing after 24 h. ### *p* < 0.001 vs. basal condition, *** *p* < 0.001 vs. TGFβ1 (Tukey’s test). SEM, standard error of the mean; sGC, soluble guanylate cyclase; TGFβ1, transforming growth factor β1.

Stimulation of human conjunctival fibroblasts with TGFβ1 significantly augmented wound-healing ability due to both cell proliferation and migration, which led to ~95% wound closure after 24 h (Figure 3). Preincubation with BAY 41-2272 10 μM resulted in a reduction of TGFβ1-mediated effects, significantly lowering wound-closure percentage up to ~65% (Figure 3).

### 3.4. TGFβ1-Induced Migration and Invasiveness of Human Conjunctival Fibroblasts Is Reduced by Treatment with BAY 41-2272

**Figure 4:**
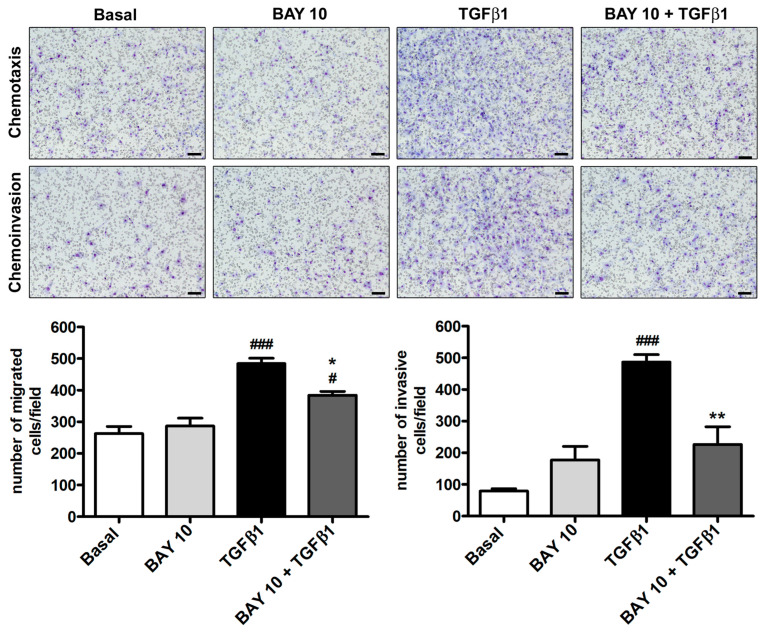
The sGC stimulator BAY 41-2272 significantly reduces TGFβ1-induced migration and invasiveness of human conjunctival fibroblasts. Representative images of the filters with migrated (chemotaxis) and invasive (chemoinvasion) cells stained with Diff-Quik are shown. Scale bar: 100 μm. Histograms represent the results of quantitative analysis of chemotaxis and chemoinvasion expressed as the number of migrated or invasive cells per field. Data are the means ± SEM of three independent experiments performed in duplicate with three cell lines. ### *p* < 0.001 and # *p* < 0.05 vs. basal condition, ** *p* < 0.01 and * *p* < 0.05 vs. TGFβ1 (Tukey’s test). SEM, standard error of the mean; sGC, soluble guanylate cyclase; TGFβ1, transforming growth factor β1.

Human conjunctival fibroblasts’ capability to migrate and invade a polymeric ECM (i.e., Geltrex) was evaluated by means of the Boyden chamber method. As presented in Figure 4, with respect to the basal condition, neither cell migration nor invasiveness were affected by administration of BAY 41-2272 10 μM alone to conjunctival fibroblasts, while they significantly increased upon stimulation with TGFβ1. Notably, cell preincubation with BAY 41-2272 was able to significantly lessen the number of both migrated and invasive conjunctival fibroblasts (Figure 4).

### 3.5. TGFβ1-Mediated Acquisition of a Myofibroblast-Like Profibrotic and Contractile Phenotype by Human Conjunctival Fibroblasts Is Attenuated by Pretreatment with BAY 41-2272

**Figure 5:**
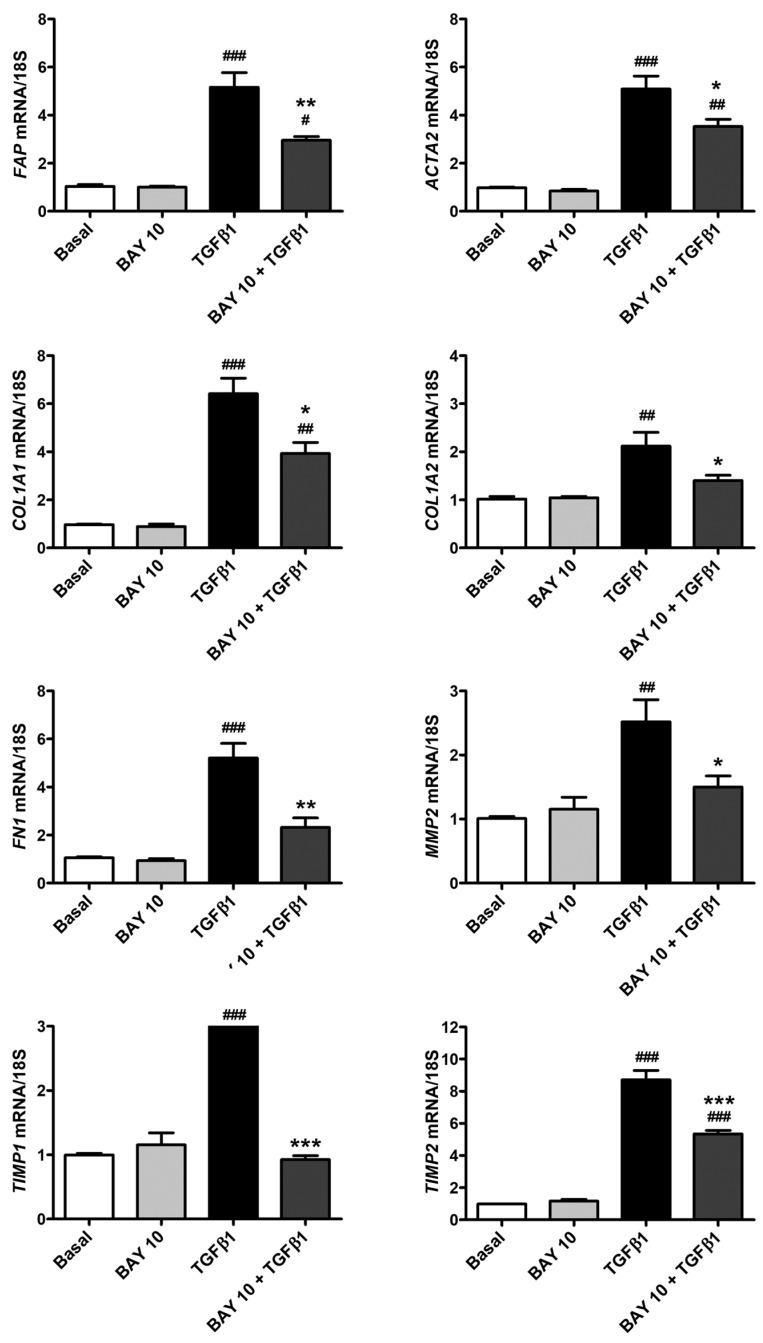
The sGC stimulator BAY 41-2272 significantly decreases TGFβ1-induced expression of genes encoding profibrotic activation/myofibroblast markers in human conjunctival fibroblasts. Gene expression of *FAP* (gene encoding fibroblast activation protein), *ACTA2* (gene encoding α-smooth muscle actin), *COL1A1* (gene encoding type I collagen α-1 chain), *COL1A2* (gene encoding type I collagen α-2 chain), *FN1* (gene encoding fibronectin 1), *MMP2* (gene encoding matrix metalloproteinase-2), *TIMP1* (gene encoding tissue inhibitor of metalloproteinases (TIMP)-1), and *TIMP2* (gene encoding TIMP-2) was measured by real-time quantitative PCR. For each gene, basal expression was set to 1, and the other results were normalized to this value. 18S ribosomal RNA was used as a reference gene. Histograms represent the mean ± SEM of triplicate determinations from three cell lines. ### *p* < 0.001, ## *p* < 0.01, and # *p* < 0.05 vs. basal condition, *** *p* < 0.001, ** *p* < 0.01, and * *p* < 0.05, vs. TGFβ1 (Tukey’s test). SEM, standard error of the mean; sGC, soluble guanylate cyclase; TGFβ1, transforming growth factor β1.

Quantitative real-time PCR performed on human conjunctival fibroblasts challenged with TGFβ1 alone showed a significant increase in gene expression of *FAP*, *ACTA2*, *COL1A1*, *COL1A2*, *FN1*, *MMP2*, *TIMP1*, and *TIMP2* (Figure 5). Notably, cell pretreatment with the sGC stimulator BAY 41-2272 10 μM significantly lowered TGFβ1-induced mRNA levels of all the abovementioned genes (Figure 5).

**Figure 6:**
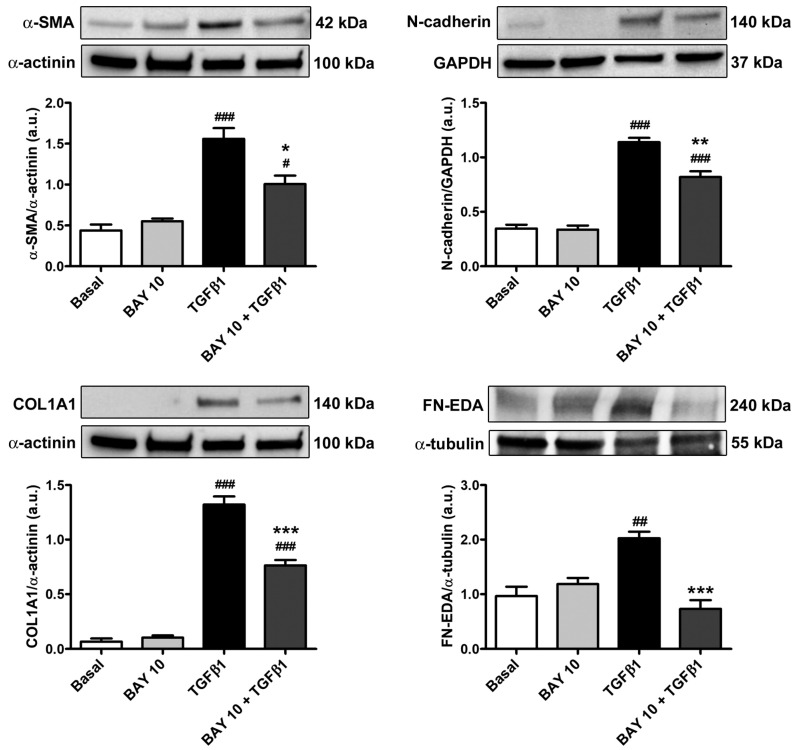
The sGC stimulator BAY 41-2272 significantly reduces TGFβ1-induced protein expression of profibrotic activation/myofibroblast markers in human conjunctival fibroblasts. Representative immunoblots for α-SMA, N-cadherin, COL1A1, and FN-EDA. For normalization, α-actinin, GAPDH, or α-tubulin were used as loading controls. The molecular weight (kDa) of each protein is shown. Histograms represent the mean ± SEM of optical density in arbitrary units (a.u.). ### *p* < 0.001, ## *p* < 0.01, and # *p* < 0.05 vs. basal condition, *** *p* < 0.001, ** *p* < 0.01, and * *p* < 0.05 vs. TGFβ1 (Tukey’s test). α-SMA, α-smooth muscle actin; COL1A1, α-1 chain of type I collagen; FN-EDA, fibronectin containing extra domain A; GAPDH, glyceraldehyde 3-phosphate dehydrogenase; SEM, standard error of the mean; sGC, soluble guanylate cyclase; TGFβ1, transforming growth factor β1.

Such findings were further strengthened by Western blotting analysis, which revealed a significant increase in the protein expression levels of both α-SMA and COL1A1 in TGFβ1-stimulated conjunctival fibroblasts (Figure 6). Interestingly, a similar significant increment was found for the myofibroblast markers N-cadherin and fibronectin containing extra domain A (FN-EDA) (Figure 6). The TGFβ1-mediated upregulation of all of these markers of activated fibroblasts/myofibroblasts was significantly dampened in conjunctival fibroblasts pretreated with BAY 41-2272 10 μM (Figure 6).

**Figure 7:**
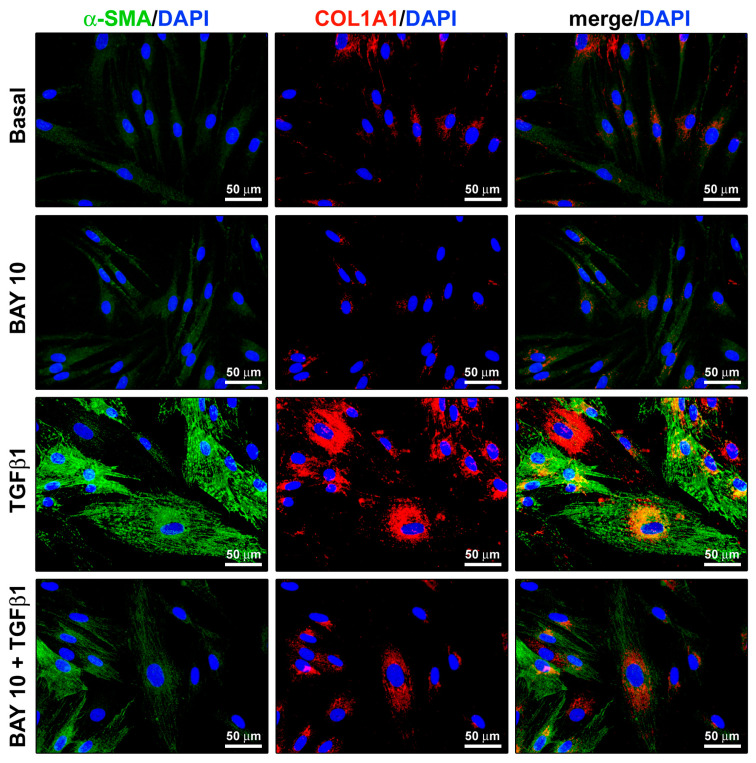
The sGC stimulator BAY 41-2272 significantly diminishes TGFβ1-induced α-SMA expression and incorporation into stress fibers and COL1A1 synthesis in human conjunctival fibroblasts. Representative fluorescence photomicrographs of cells immunostained for α-SMA (green) and COL1A1 (red) are shown. Nuclei are counterstained with DAPI (blue). Scale bar = 50 μm. α-SMA, α smooth muscle actin; COL1A1, α-1 chain of type I collagen; DAPI, 4′,6-diamidino-2-phenylindole; sGC, soluble guanylate cyclase; TGFβ1, transforming growth factor β1.

The capability of the BAY 41-2272 compound to attenuate TGFβ1-induced α-SMA and COL1A1 expression was additionally confirmed by fluorescence immunocytochemistry (Figure 7). In particular, both α-SMA immunopositivity and incorporation into stress fibers resulted strongly reduced when conjunctival fibroblasts were administered with BAY 41-2272 before stimulation with TGFβ1 (Figure 7).

**Figure 8:**
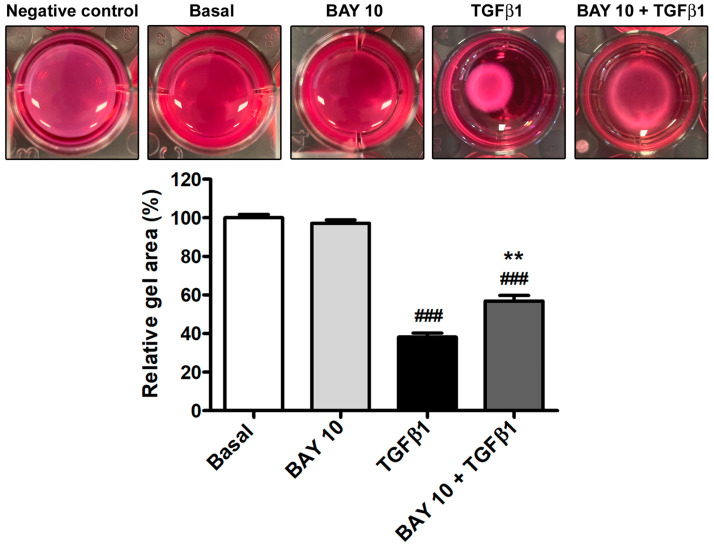
The sGC stimulator BAY 41-2272 significantly dampens the TGFβ1-induced contractile ability of human conjunctival fibroblasts. Representative wells of the collagen gel contraction assay are shown. Every experimental point was performed in triplicate. Histograms represent the mean ± SEM of gel sizes expressed as the percentage of those observed in wells with cells at basal condition. ### *p* < 0.001 vs. basal condition, ** *p* < 0.01 vs. TGFβ1 (Tukey’s test). SEM, standard error of the mean; sGC, soluble guanylate cyclase; TGFβ1, transforming growth factor β1.

In agreement with the findings on α-SMA protein expression and arrangement, cell preincubation with BAY 41-2272 10 μM also resulted in a significant lessening of the TGFβ1-induced capability of conjunctival fibroblasts to contract a collagen gel matrix (Figure 8).

**Figure 9:**
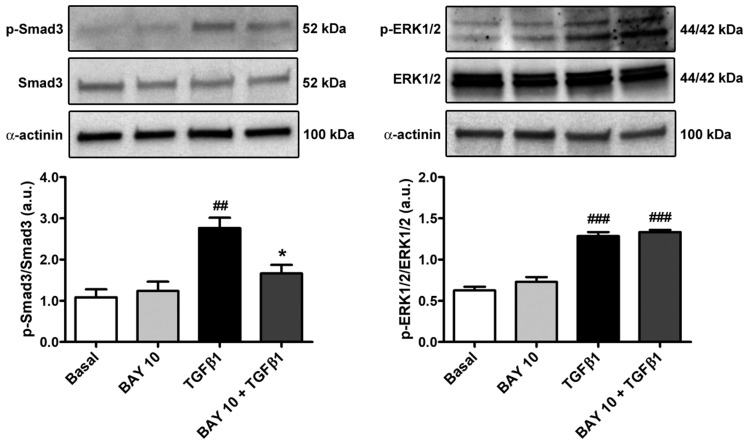
The sGC stimulator BAY 41-2272 significantly reduces TGFβ1-induced Smad3-dependent canonical TGFβ1 signaling in human conjunctival fibroblasts. Representative immunoblots for p-Smad3, total Smad3, p-ERK1/2, and total ERK1/2 are shown. α-actinin was used as a loading control for normalization. The molecular weight (kDa) of each protein is shown. Bars represent the mean ± SEM of optical density in arbitrary units (a.u.). ### *p* < 0.001 and ## *p* < 0.01 vs. basal condition, * *p* < 0.05 vs. TGFβ1 (Tukey’s test). p-ERK1/2, phosphorylated-ERK1/2; p-Smad3, phosphorylated-Smad3; SEM, standard error of the mean; sGC, soluble guanylate cyclase; TGFβ1, transforming growth factor β1.

Considering that Smad3 phosphorylation and ERK1/2 phosphorylation represent important steps of the TGFβ1-mediated canonical and non-canonical pathways, respectively, we next evaluated the protein levels of both phosphorylated-Smad3/total Smad3 and phosphorylated-ERK1/2/total ERK1/2 in the different experimental conditions. Conjunctival fibroblast stimulation with TGFβ1 alone significantly augmented both Smad3 and ERK1/2 protein phosphorylation (Figure 9). Of note, cell pretreatment with BAY 41-2272 10 μM significantly lessened only phosphorylated-Smad3, reaching levels comparable to those of the basal condition (Figure 9).

### 3.6. BAY 41-2272 Administration Lessens TGFβ1-Induced Secretion of Proinflammatory Cytokines by Human Conjunctival Fibroblasts

**Figure 10:**
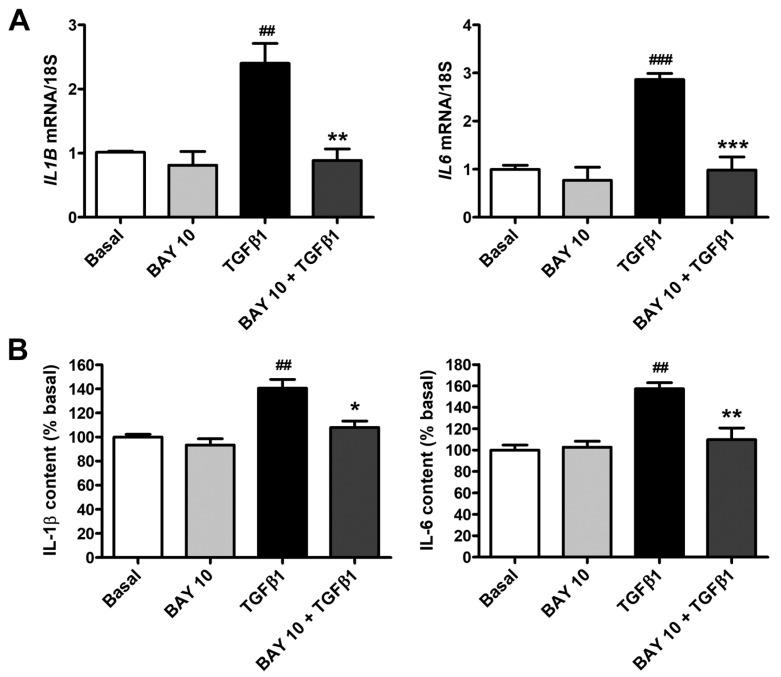
The sGC stimulator BAY 41-2272 significantly lowers TGFβ1-induced gene expression and secretion of proinflammatory cytokines in human conjunctival fibroblasts. (**A**) Gene expression of *IL1B* (gene encoding IL-1β) and *IL6* (gene encoding IL-6) was evaluated by real-time quantitative PCR. 18S ribosomal RNA was used as a reference gene. The basal level of each gene was set to 1, and the other results were normalized to this value. (**B**) Protein levels of IL-1β and IL-6 were measured in culture supernatants by enzyme-linked immunosorbent assay and expressed as a percentage of those at basal condition. Bars represent the mean ± SEM of triplicate determinations from three cell lines. ### *p* < 0.001 and ## *p* < 0.01 vs. basal condition, *** *p* < 0.001, ** *p* < 0.01, and * *p* < 0.05 vs. TGFβ1 (Tukey’s test). IL, interleukin; SEM, standard error of the mean; sGC, soluble guanylate cyclase; TGFβ1, transforming growth factor β1.

As reported in Figure 10A, TGFβ1 stimulation determined a strong upregulation of both *IL1B* and *IL6* mRNA levels, an effect that was significantly lowered by BAY 41-2272 10 μM pretreatment. In agreement with these results, IL-1β and IL-6 levels were found to be significantly augmented in the supernatants of TGFβ1-treated conjunctival fibroblasts and resulted strongly decreased in cells pretreated with BAY 41-2272 10 μM (Figure 10B). In particular, the levels of IL-1β and IL-6 secreted by conjunctival fibroblasts pretreated with BAY 41-2272 and then stimulated with TGFβ1 were both like those measured in the supernatants of basal cells (Figure 10B).

## 4. Discussion

In this study, we investigated, for the first time, the in vitro effects of pharmacological stimulation of the sGC enzyme on the profibrotic activation of human conjunctival fibroblasts. Our data clearly prove that the administration of the sGC stimulator BAY 41-2272 is effective in reducing TGFβ1-induced conjunctival fibroblast proliferation, migration, invasiveness, transition to profibrotic myofibroblasts, and secretion of proinflammatory cytokines.

Conjunctival fibrosis is a serious clinical concern implicated in a wide spectrum of ocular diseases, and in the outcomes of ocular surgery for pterygium and glaucoma, and is mainly driven by chronic inflammation, with the recruitment of mononuclear cells releasing several profibrotic cytokines such as TGFβ [3,6,7,9,10,39]. In such a pathologic microenvironment, conjunctival fibroblasts are stimulated to proliferate, migrate, and produce ECM, finally differentiating into myofibroblasts and leading to abnormal wound healing and scar formation [3,6,7,9,10,39]. In glaucoma, excessive scarring of the filtering bleb is the primary cause for the failure of filtration surgery, with scar formation causing complications such as unstable intraocular pressure and decreased vision [5,19,20]. Conjunctival inflammation that triggers an undesired fibrogenic process contributing to conjunctival scarring is also frequently associated with toxic or allergic responses to the preservatives contained in long-term topical glaucoma medication [10,14].

The TGFβ family comprises three isoforms (namely TGFβ1, TGFβ2, and TGFβ3), all exerting biological effects through the same receptors. Despite the preponderance of TGFβ2 in the vitreous and aqueous humor, as well as in tears in several eye pathologies [17,23], the TGFβ1 isoform has been found to be overexpressed in pterygium conjunctiva, conjunctival fibroblasts explanted from recurrent pterygia, and the aqueous humor of individuals with clinically active glaucoma [40], where it was reported to be a strong inducer of conjunctival myofibroblast activation [15,19,20,21,22,24,41,42,43]. Moreover, at variance with TGFβ2, TGFβ1 was found to powerfully increase stiffness in human conjunctival fibroblast spheroids, and ECM stiffness is well known to foster the differentiation of fibroblasts toward a profibrotic myofibroblastic phenotype [23]. For these reasons, we chose the TGFβ1 as the inducer of conjunctival fibroblast transition to profibrotic myofibroblasts in our in vitro experimental setting.

A growing body of literature has demonstrated that increased cGMP levels due to sGC enzyme stimulation/activation may suppress TGFβ-mediated myofibroblast differentiation in various stromal cells, such as human skin and pulmonary fibroblasts [27,33,34,35], and inhibit both dermal endothelial- and renal epithelial-to-mesenchymal transitions [36,44]. Interestingly, the contribution of sGC stimulation and the cGMP pathway in preventing myofibroblast differentiation has been also recently demonstrated in cultures of human keratocytes stimulated with TGFβ1 [37,45]. Based on this background, we sought to assess the impact of pharmacological stimulation of the sGC enzyme on TGFβ1-induced transdifferentiation of human conjunctival fibroblasts into myofibroblasts. As we have already established for human primary corneal keratocytes [37] and in agreement with the abovementioned study by Park et al., in which no toxic effect was noticed for exogenous NO as an sGC activator [45], we herein demonstrated that sGC stimulation with the BAY 41-2272 compound tested at two concentrations (1 µM and 10 µM) did not affect human conjunctival fibroblast viability, thus representing its potential suitability as an ophthalmic medication. In addition, we assessed that sGC stimulation is able to significantly counteract TGFβ1-induced conjunctival fibroblast proliferation, as already reported for both human keratocytes and pulmonary fibroblasts [34,37]. When comparing the effects of the aforementioned BAY 41-2272 concentrations, we found that the higher dose (10 µM) was more effective in dampening TGFβ1-induced cell proliferation; therefore, for all of our subsequent experiments, we employed only this concentration of this pharmacological compound. In addition to enhancing proliferation, TGFβ1 is well known to make conjunctival fibroblasts prone to acquiring a profibrotic myofibroblast-like phenotype by inducing morphological changes, increasing their migratory and invasive abilities, and promoting their contractile properties [8,15,19,21,22,24,41,46]. In our study, TGFβ1-treated cells showed larger dimensions and highly flattened and polygonal-shaped cytoplasm due to cytoskeletal rearrangement and vinculin-positive focal adhesion formation, all morphological changes that were significantly dampened by preincubation with BAY 41-2272. The ability of sGC stimulation to reduce both cell proliferation and size was further confirmed by evidence that cell confluency percentage was comparable to that of the basal condition in TGFβ1-treated cells that were preincubated with BAY 41-2272. We also demonstrated that the sGC stimulator BAY 41-2272 significantly prevented TGFβ1-triggered excessive wound-healing capacity and invasiveness, and lowered the synthesis of COL1A1 and the expression of a variety of myofibroblast markers, such as N-cadherin, FN-EDA, and α-SMA [47,48], ultimately leading to diminished contractile ability. These findings are in accordance with two previous studies reporting that both indirect and direct sGC activation through NO and BAY 41-2272 compound, respectively, significantly attenuated TGFβ1-driven myofibroblastic differentiation in human keratocytes [37,45]. Moreover, the sGC stimulator BAY 41-2272 was found to significantly dampen TGFβ1-mediated proliferation, collagen and fibronectin production, and α-SMA rearrangement in contractile fibrils in healthy human lung fibroblasts [27,33,34]. The stimulation/activation of sGC was also shown to significantly inhibit the myofibroblastic differentiation of human prostatic and dermal fibroblasts, as well as to blunt myofibroblast-like features of endothelial cells harvested from fibrotic skin lesions of patients with systemic sclerosis [27,33,35,36].

The altered activation of matrix metalloproteinases (MMPs) and tissue inhibitor of metalloproteinases (TIMPs) is also known to influence ECM composition in the trabecular meshwork and to contribute to the onset and progression of glaucoma [49]. In addition, a marked increase in MMP-2 and TIMP-2 levels accompanied by the development of a scarring process has been observed in a rat conjunctival bleb model [50], while in the aqueous humor of individuals with acute primary angle closure, higher MMP-2 and TIMP-1 levels were reported to correlate with excessive ECM deposition [49]. Interestingly, in our study, sGC stimulation with the BAY 41-2272 compound was effective in counteracting the upregulation of *MMP2*, *TIMP1*, and *TIMP2* gene expression levels in human conjunctival fibroblasts treated with TGFβ1, suggesting that such a pharmacological approach may hamper conjunctival fibrosis by controlling the expression of a wide array of molecules involved in ECM remodeling.

Recently published works have reported that Smad-mediated canonical signaling is the main pathway underlying the TGFβ1-mediated transdifferentiation of conjunctival and corneal fibroblasts into myofibroblasts [8,15,24,41,51,52]. As far as pharmacological sGC stimulation is concerned, it has been found to inhibit the ERK1/2-mediated non-canonical TGFβ signaling pathway in human skin fibroblasts and murine experimental models of cutaneous fibrosis [28,33]. Interestingly, we found that pharmacological stimulation of the sGC enzyme was able to inhibit only the canonical TGFβ pathway in human conjunctival fibroblasts, as BAY 41-2272 administration did not change the levels of phosphorylated-ERK1/2 but significantly decreased TGFβ1-induced Smad3 phosphorylation. These results are in line with a recent study from our group, in which stimulation of sGC with the same pharmacological compound was able to counteract the TGFβ1-induced profibrotic behavior of human keratocytes by decreasing phosphorylated-Smad3 protein levels [37].

It is known that the inflammatory pathological milieu consequent to both glaucoma surgery wound and topical long-term glaucoma treatment further induces conjunctival fibroblasts to secrete various growth factors and proinflammatory cytokines, such as IL-1β and IL-6 [11,53]. Here, we demonstrated that, besides showing antifibrotic effects, the sGC stimulator BAY 41-2272 exerted anti-inflammatory properties, being capable of diminishing the TGFβ1-induced acquisition of a proinflammatory phenotype by human conjunctival fibroblasts through a significant reduction in the secretion of IL-1β and IL-6. Consistent with these findings, previous studies have reported the anti-inflammatory effects of pharmacological stimulation with sGC enzyme in various experimental models of tissue fibrosis and inflammation, such as non-alcoholic steatohepatitis and the Dahl model of cardiorenal failure [54,55,56].

At present, clinical treatment of conjunctival fibrotic conditions, mainly scarring after glaucoma filtration surgery or post-operative long-term use of topical medications, is relatively limited and includes antiscarring and antimetabolite drugs such as mitomycin-C and 5-fluorouracil [3,57]. However, the topical application of these therapeutics is restricted due to their toxic effects and the occurrence of adverse reactions that can potentially lead to blindness [3,57]. Therefore, there is an urgent need to identify new therapeutic strategies to inhibit fibroblast differentiation into myofibroblasts and excessive ECM synthesis, consequently alleviating conjunctival fibrosis.

## 5. Conclusions

Collectively, our data demonstrate the efficacy of the BAY 41-2272 compound in counteracting human conjunctival fibroblast-to-myofibroblast transition, thus paving the way for the potential employment of sGC stimulators as new adjuvant therapeutic agents for the treatment of conjunctival fibrotic conditions such as in pterygium or glaucoma. Of note, recent research has suggested that cGMP signaling may play a direct neuroprotective role for retinal ganglion cells, whose degeneration is responsible for progressive vision loss in glaucoma [58]. On these bases, it is tempting to speculate that pharmacological stimulation of sGC might represent an innovative therapeutic strategy for the management of both glaucoma-related fibrosis and neurodegenerative changes. However, as we performed only in vitro experiments herein, further preclinical studies will be required to extend our observations and confirm the safety and efficacy of topical ophthalmic administration of BAY 41-2272 in vivo. Indeed, an in vivo approach will allow a more comprehensive assessment of the impact of sGC stimulation with BAY 41-2272 in the context of conjunctival tissue heterogeneity, such as any effect on conjunctival epithelial cells and inflammatory/immune cells. In addition, since we focused on the ability of sGC stimulation to prevent TGFβ1-induced profibrotic effects on conjunctival fibroblasts in the present study, it will be interesting to investigate whether the same approach may also revert the fibroblast-to-myofibroblast transition. Finally, it is important to highlight that besides sGC stimulators, another class of compounds capable of activating the sGC enzyme is represented by sGC activators, which specifically activate dysfunctional, oxidized, and heme-free sGC enzyme [25]. Among them, the sGC activator MGV354 has been demonstrated to lower intraocular pressure in preclinical models of glaucoma, though a subsequent clinical trial failed to produce similar results in patients with ocular hypertension or open-angle glaucoma [59,60,61]. Nevertheless, it remains to be ascertained if sGC activators may exert similar effects as BAY 41-2272 on conjunctival fibroblasts, or even more powerful antifibrotic effects, especially in conditions of oxidative stress. In such a context, we are confident that the present findings represent the essential basis for future preclinical research on the feasibility of pharmacological sGC stimulation/activation as a potential new therapeutic approach against conjunctival fibrosis, which complicates a variety of ocular disorders.

## Figures and Tables

**Table 1:** Predesigned oligonucleotide primer pairs used for SYBR Green real-time PCR.

Gene	Assay ID	Catalog Number
*FAP*	Hs_FAP_1_SG	QT00074963
*ACTA2*	Hs_ACTA2_1_SG	QT00088102
*COL1A1*	Hs_COL1A1_1_SG	QT00037793
*COL1A2*	Hs_COL1A2_1_SG	QT00072058
*FN1*	Hs_FN1_1_SG	QT00038024
*MMP2*	Hs_MMP2_1_SG	QT00088396
*TIMP1*	Hs_TIMP1_1_SG	QT00084168
*TIMP2*	Hs_TIMP2_1_SG	QT00017759
*IL1B*	Hs_IL1B_1_SG	QT00021385
*IL6*	Hs_IL6_1_SG	QT00083720

**Table 2:** Details of primary antibodies used for Western blotting analysis.

Primary Antibody	Host Species	Catalog Number	Producer	Dilution
anti-α-SMA	Mouse	ab7817	Abcam, Cambridge, UK	1:300
anti-N-cadherin	Rabbit	#13116S	Cell Signaling Technology, Danvers, MA, USA	1:1000
anti-COL1A1	Rabbit	#39952	Cell Signaling Technology, Danvers, MA, USA	1:1000
anti-fibronectin	Mouse	SAB4200880	Sigma-Aldrich, St. Louis, MO, USA	1:1000
anti-Smad3	Rabbit	#9513S	Cell Signaling Technology, Danvers, MA, USA	1:1000
anti-p-Smad3	Rabbit	#9520S	Cell Signaling Technology, Danvers, MA, USA	1:1000
anti-ERK1/2	Rabbit	ab17942	Abcam, Cambridge, UK	1:1000
anti-p-ERK1/2	Goat	sc-16982	Santa Cruz Biotechnology, Dallas, TX, USA	1:1000
anti-α-actinin	Rabbit	#3134	Cell Signaling Technology, Danvers, MA, USA	1:1000
anti-GAPDH	Mouse	ab8245	Abcam, Cambridge, UK	1:5000
anti-α-tubulin	Rabbit	#2144	Cell Signaling Technology, Danvers, MA, USA	1:1000

α-SMA, anti-α-smooth muscle actin; p-Smad3, phosphorylated-Smad3 (Ser423/425); p-ERK1/2, phosphorylated-ERK1/2 (Thr202/Tyr204); GAPDH, glyceraldehyde 3-phosphate dehydrogenase.

## Data Availability

All relevant data are included within the manuscript.

## Supplementary Materials

The following supporting information can be downloaded at https://www.mdpi.com/article/10.3390/cells13040360/s1, Figure S1: Intracellular levels of cGMP in human conjunctival fibroblasts.
