# Supplementary material for: Pharmacological Stimulation of Soluble Guanylate Cyclase Counteracts the Profibrotic Activation of Human Conjunctival Fibroblasts
Source: Cells. 2024 Feb 18;13(4):360. doi: 10.3390/cells13040360 (PMC10887040; doi:10.3390/cells13040360)
Supplement: Supplementary file 1 [file cells-13-00360-s001.zip › cells-2863216-supplementary.pdf]

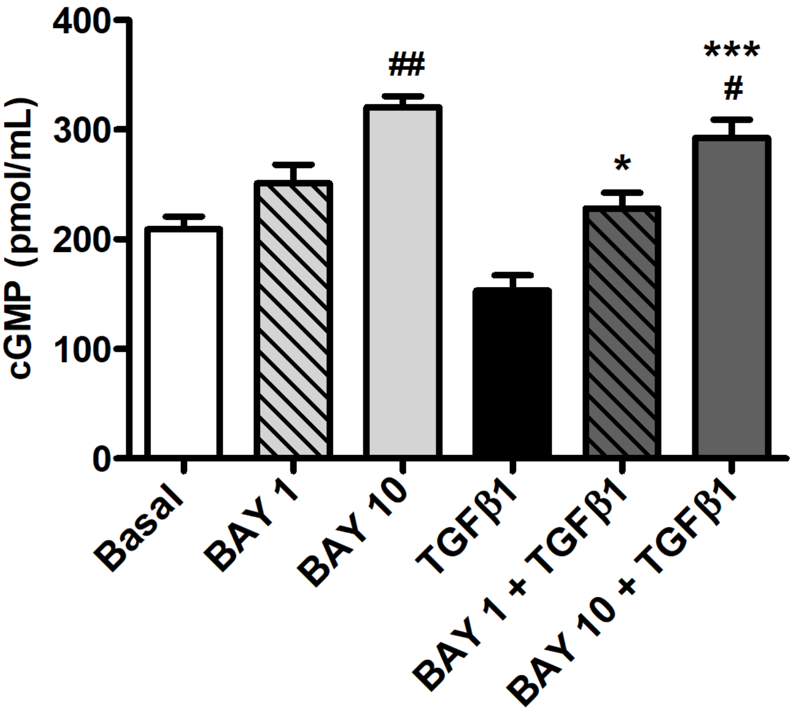

Supplementary Figure S1. Intracellular levels of cGMP in human conjunctival fibroblasts. Stimulation of sGC with BAY 41-2272 effectively increases the intracellular levels of cGMP in human conjunctival fibroblasts. Bars represent the mean ± SEM of triplicate determinations from three cell lines. ## $p < 0.01$ and # $p < 0.05$ vs. basal condition, *** $p < 0.001$ and * $p < 0.05$ vs. TGFβ1 (Tukey's test). cGMP, cyclic guanosine monophosphate; SEM, standard error of the mean; sGC, soluble guanylate cyclase; TGFβ1, transforming growth factor β1.
